# Secretion of *Bacillus amyloliquefaciens* Transglutaminase from *Lactococcus lactis* and Its Enhancement of Food Gel Properties

**DOI:** 10.3390/gels8100674

**Published:** 2022-10-20

**Authors:** Tiange Ma, Xingjiang Li, Manuel Montalbán-López, Xuefeng Wu, Zhi Zheng, Dongdong Mu

**Affiliations:** 1 Anhui Fermented Food Engineering Research Center, School of Food and Biological Engineering, Hefei University of Technology, Hefei 230601, China; 2 Department of Microbiology, Faculty of Sciences, University of Granada, 18071 Granada, Spain; 3 School of Food and Biological Engineering, Key Laboratory for Agricultural Products Processing of Anhui Province, Hefei University of Technology, Hefei 230601, China

**Keywords:** *Lactococcus lactis*, microbial transglutaminase (MTGase), SPI gel

## Abstract

(1) Background: Microbial transglutaminases (MTGase) catalyze protein crosslink. This is useful in the food industry to improve gelation, water holding capacity, and emulsifying capacity during foodstuff manufacturing. The production of MTGase in wild-type strains renders low yield and high costs of downstream purification, limiting its industrial applications. (2) Methods: In this work, MTGase from *Bacillus amyloliquefaciens* BH072 (BaMTGase) has been heterologously expressed in *Lactococcus lactis*, using the signal peptide Usp45 to direct the secretion of recombinant BaMTGase out of the cell for easier purification. (3) Results: In these conditions, MTGase was purified with a high yield (48.7 ± 0.2 mg/L) and high enzyme activity (28.6 ± 0.5 U/mg). Next, BaMTGase was tested for industrial applications. Recombinant BaMTGase and commercial MTGase were used for SPI solution crosslinking. BaMTGase formed a harder gel with higher water-holding capacity and a dense and smooth gel microstructure. (4) Conclusions: This work provides an attractive food-grade cell factory for the food industry and offers a suitable chassis for MTGase production.

## 1. Introduction

Microbial transglutaminases (EC 2.3.2.13, MTGase) catalyze acyl-transfer reactions between γ-carboxamide groups of glutamine residues and ε-amino groups of lysine residues. In this way, they create inter- or intramolecular covalent crosslinks consisting of ε-(γ-glutamyl)-lysine isopeptide bonds [1,2,3]. In the food industry (including the processing of dairy products, meat products, soy/wheat products, and others), MTGase has considerable potential for application, because MTGase does not destroy the nutritional quality of food and the reaction not only enhances the properties of the protein, such as solubility, emulsifying properties, viscoelasticity, gelation and water holding capacity (WHC), but also improves the taste, texture and shelf life [4,5,6]. MTGase is recognized as a food additive that adheres to the GRAS (Generally Recognized as Safe) standard by FDA (Food and Drug Administration) since 1998. In addition, the process of crosslinking may introduce essential amino acids to make food more nutritious [7,8].

Transglutaminases (TGase) are present in animal tissues, plants, and microorganisms. Direct extraction of TGase from animal and plant tissues is costly, it is dependent on Ca^2+^ and cannot be directly applied to the food industry [9]. MTGases are Ca^2+^ independent enzymes that exhibit broad substrate tolerance and can catalyze the reaction over a wide range of pH and temperatures [10]. Ando et al. isolated the MTGase from *Streptomyces mobaraensis* in soil and produced MTGase by traditional fermentation technology [11]. Nonetheless, the yield and enzyme activity of MTGase from the wild-type strain was low for downstream applications. Nowadays, commercial MTGase is mainly produced by fermentation using the wild-type *S. mobaraensis* strain [12]. The fermentation process is complex and energy sources are scarce, there is a loss of yield due, for instance, to MTGase hydrolyzation by extracellular proteases [4]. Inexpensive commercial MTGase in the market contains impurities, while pure commercial MTGase has a high price. With the development of biotechnology, the focus of research has shifted to genetic engineering (constructed engineered strains) to improve the yield and enzymatic properties of MTGase [13,14]. To solve the problem mentioned above, researchers turned to genetic engineering technology to search for alternative microbiological sources, easy to manipulate and that can render high yield and purity in a cost-effective manner [14,15,16].

Diverse food-grade cell factories that increase the quality and safety of food products have been established. To date, Lactic acid bacteria (LAB) and *Bacillus subtilis* are the main food-grade prokaryotic microorganisms used in the production of commercially relevant secreted proteins [17]. Because *Lactococcus lactis* (*L. lactis*) have a simple metabolism, relatively small genome, low extracellular protease activity, and absence of endotoxins, these advantages make *L. lactis* to be an attractive cell factory to produce heterologous proteins [15,18]. In *Lactococcus lactis* NZ9000 (*L. lactis* NZ9000), the nisin-inducible controlled gene expression (NICE) system was established by the team of O. P. Kuipers, so that the precise regulation of protein production is controlled [19]. Protein secretion enables easy purification of heterologously produced enzymes, therefore decreasing the costs. In *L. lactis*, the Usp45-signal peptide is the most widely used signal peptide (SP) for the secretion of heterologous proteins [20]. In this work, we constructed a heterologous MTGase production system transferring the *Bacillus amyloliquefaciens* BH072 transglutaminase (BaMTGase) gene to the *L. lactis* NZ9000. Engineering a suitable secretion signal allowed the purification of active recombinant BaMTGase whose properties were further investigated.

## 2. Results

### 2.1. Results Analysis

#### 2.1.1. Secretion of Heterologous BaMTGase in *L. lactis* NZ9000

**Figure 1:**
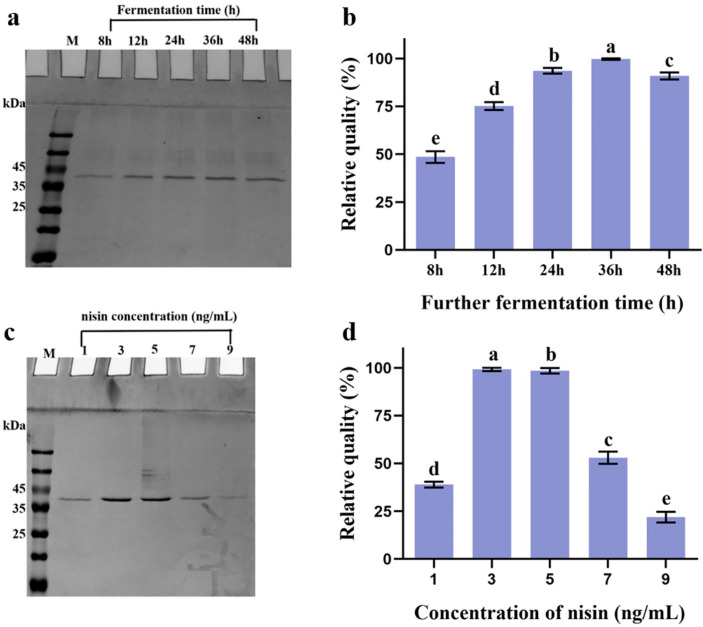
Expression of pro-BaMTGase and optimization of fermentation conditions. M: molecular weight marker. (**a**) Optimization of fermentation time. 1 ng/mL nisin was added when the strain reached the logarithmic phase, and the culture continued for another 8 h, 12 h, 24 h, 36 h, and 48 h. (**b**) Relative quantities of SPUsp45-pro-BaMTGase produced by *L. lactis* NZ9000 (pNZ8048-SP*usp45-pro-Bamtg*) induced with 1 ng/mL nisin. (**c**) Optimization of inducer concentration. The expression of pro-BaMTGase after induction for 36 h with 1, 3, 5, 7, 9 ng/mL nisin. (**d**) Relative expression quantities of SPUsp45-pro-BaMTGase in *L. lactis* NZ9000 (pNZ8048-SP*usp45-pro-Bamtg*) induced with nisin for 36 h at different concentrations. a–e above the bars in (**b**,**d**) represent the significant differences (*p* < 0.05).

To obtain BaMTGase into the medium, the DNA encoding Usp45 signal peptide (SP*usp45*) was fused to the *B. amyloliquefaciens mtg* gene to direct the secretion. Suitable restriction sites and a C-terminal 6-His were added to enable easy cloning and enzyme purification. As shown in Figure 1a, *L. lactis* (pNZ8048-SP*usp45-pro-Bamtg*) could express and secrete heterologous BaMTGase under the control of nisin. When the OD_600_ reached 0.6, 1 ng/mL nisin was added and the incubation was prolonged for 8 h, 12 h, 24 h, 36 h, and 48 h. Bands corresponding to the propeptide BaMTGase (Pro-BaMTGase, 36.4 kDa), containing the SPUsp45-proregion (7.3 kDa). According to BANDSCAN analysis, the highest yield was obtained at 36 h after adding nisin, with a concentration of 40.3 ± 0.4 mg/L. This was 2/1.3/1/1.1 times higher than for the other fermentation times (8 h/12 h/24 h/48 h, respectively, *p* < 0.05) (Figure 1b). Protein expression is also dependent on the concentration of the inducer; therefore, the amount of inducer was investigated to raise the yield of BaMTGase. After analysis by SDS-PAGE as well as BANDSCAN software, no linear relationship between the change in inducer concentration was found. The yield of protein was optimal at 3 ng/mL nisin (Figure 1c,d). Using 3 ng/mL nisin and 36 h induction 48.7 ± 0.2 mg/L pro-BaMTGase was obtained, thus the relative concentration was 1.2 times higher than with 1 ng/mL inducer. As shown in Figure S2, we found that the protein secretion had little effect on the growth of the strains, although there was a backward shift in the time to reach the logarithmic phase, the absorbance of the stable phase remained balanced.

#### 2.1.2. Activation of Pro-BaMTGase and Determination of Enzyme Activity

**Figure 2:**
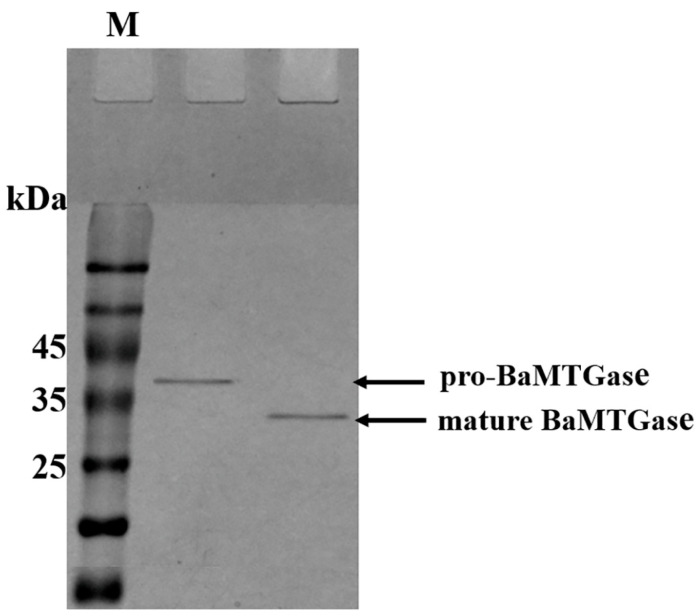
Proteolysis of SPUsp45-pro-BaMTGase with trypsin to achieve mature BaMTGase. M: molecular weight marker.

Recombinant BaMTGase was secreted to GM17 medium in the form of inactive pro-BaMTGase (the precursor peptide barely catalyzes the substrate reaction). To activate the zymogen, trypsin was used before purification to digest the signal peptide and proregion in the supernatant. After proteolysis, the proregion was eliminated. In Figure 2, the mature BaMTGase was obtained with a band size that matched the molecular weight (29.2 kDa). The purified enzyme was active with an enzymatic activity of 28.6 ± 0.5 U/mg, it was 28.6 times that of the commercial MTGase. In addition, as shown in Figure 2, there was almost no difference in gray between the two protein bands, and the concentration of the mature BaMTGase was 46.9 ± 0.8 mg/L.

#### 2.1.3. Crosslinking of 1% (*w*/*v*) SPI

**Figure 3:**
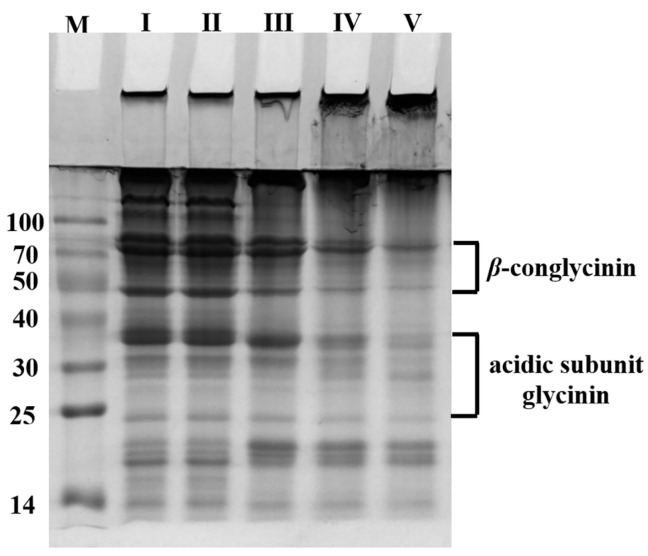
Protein crosslinking assay using 1% SPI and MTGases. Lane I: SPI solution without any treatment, considered as a control in this experiment. Lane II and lane III: the samples were crosslinked by commercial MTGase for 15 min and 60 min. Lane IV and lane V: BaMTGase crosslinked SPI solution after 15 min and 60 min.

To investigate the applicability of BaMTGase, the covalent binding of SPI protein subunits after enzymatic crosslinking was monitored by SDS-PAGE. The same concentration of mature BaMTGase and commercial MTGase were mixed with 1% (*w*/*v*) SPI solution, respectively. Figure 3 reveals the difference in crosslinking activity between commercial MTGase and mature BaMTGase. As shown in Figure 3, it was observed that BaMTGase produced an excellent crosslinking effect on SPI solution when the samples were treated at 45 °C for 60 min. At the top of stacking gel and separating gel, there was a larger accumulation of high molecular weight proteins, and the content of *β*-conglycinin and the glycinin acidic subunit visibly decreased.

#### 2.1.4. Gel Strength and Water-Holding Capacity

**Figure 4:**
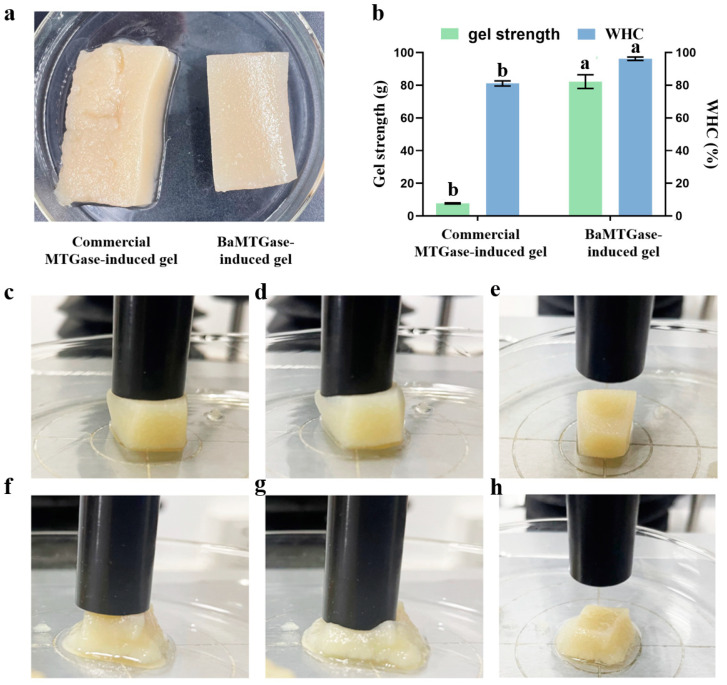
Gel strength and WHC of SPI gels. (**a**) Visual comparison of gels induced by commercial MTGase and recombinant BaMTGase. (**b**) Gel strength and WHC of SPI gels crosslinked by commercial MTGase and BaMTGase. (**c**) Morphological changes of the gel induced by BaMTGase when the texture analyzer touched the surface. (**d**) Morphological changes of BaMTGase-induced gel when the instrument squeezed gels. (**e**) Morphological changes of BaMTGase-induced gel after squeezing. (**f**) Morphological changes of commercial MTGase-induced gel when the texture analyzer touched the surface. (**g**) Morphological changes of commercial MTGase-induced gel when the instrument squeezed the gel. (**h**) Morphological changes of commercial MTGase-induced gel after squeezing. Letters above the bars in (**b**) indicate significant differences (*p* < 0.05).

Gel strength and WHC are important properties for evaluating the quality of a gel, which could reflect the interaction between proteins and water in the gel system [21]. The visual comparative plots of gel strength and WHC of SPI gels were shown in Figure 4a,b, and the textures of MTGase-induced SPI gels were listed in Table 1. The enzymatic activity of BaMTGase was 28.6 times higher than that of the commercial MTGase. Thus, although only 2 mg BaMTGase was added to the SPI solution to induce the formation of SPI gel, the gel strength and WHC of BaMTGase-induced gel were 82.3 ± 0.4 g and 96.3%. When 300 mg commercial MTGase was added to the SPI solution, the gel strength and WHC of commercial MTGase-induced sample were 7.7 ± 0.3 g and 81.1%. Meanwhile, the data presented in Table 1 suggested that BaMTGase-induced SPI gels possessed higher hardness, springiness, cohesiveness, and gumminess. This demonstrates that the commercial MTGase-induced gel was unstable.

#### 2.1.5. FT-IR Analysis

**Figure 5:**
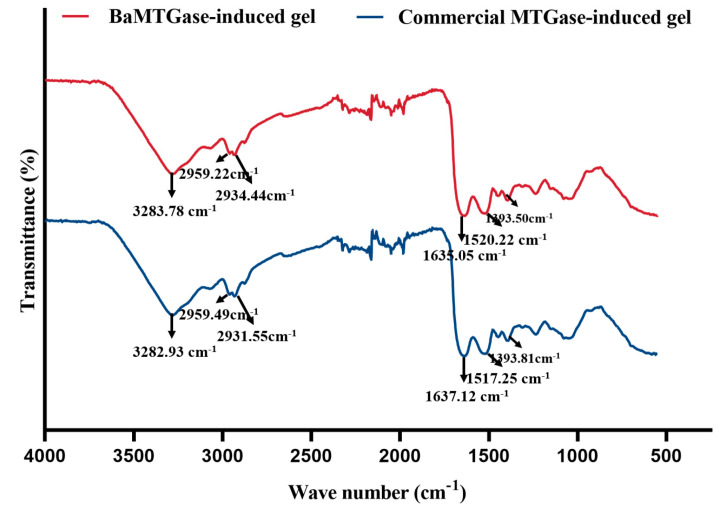
The FT−IR spectra of SPI gel.

The secondary structure of the protein could be investigated with the help of the FTIR spectrum. As shown in Figure 5, the amide I region in the 1600–1700 cm^−1^ range is often used to analyze the secondary structure of proteins. The scope of each secondary structure attribution (1650–1660 cm^−1^, *α*-helix; 1618–1640 cm^−1^ and 1670–1690 cm^−1^, *β*-sheets; 1660–1670 cm^−1^ and 1690–1700 cm^−1^, *β*-turns; 1645 cm^−1^, random coils) referred to Wang et al. [22]. The proportion of the corresponding secondary structures of MTGase-induced gels was calculated based on the area of the peaks. As the data summarized in Table 2, the β-sheet structure and *β*-turns of the BaMTGase-induced SPI gel increased and the *α*-helix structure decreased.

#### 2.1.6. Microstructure of the Gel

**Figure 6:**
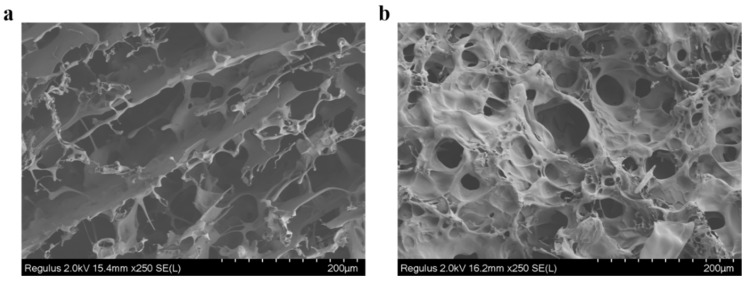
Microstructure of the SPI gel under the electron microscope. (**a**) Microstructure of the commercial MTGase-induced gel. (**b**) Microstructure of the BaMTGase-induced gel.

To visualize the changes in the SPI gels network, the microstructure of SPI gels was investigated by FESEM. As shown in Figure 6a, the structure of commercial MTGase-induced SPI gel exhibited irregular sheet-like clusters and loose and rough pores, and no obvious crosslinked network was formed inside the holes. In contrast, the structure of the BaMTGase-induced gel was homogenous, the pores became smaller and smoother, and a stable connection was formed inside the pores (Figure 6b).

### 2.2. Discussion

Microbial transglutaminase is a useful additive in the food industry. It has provided new functions for foods and reduced waste in food manufacturing [23]. By controlling the reaction conditions during fabrication, a variety of modifications can be achieved to improve the physicochemical properties of proteins. Additionally, MTGase has provided enormous contributions in the fields of biomedicine, materials science, and textiles [24,25]. Currently, the challenge of MTGase is the limited yield and enzyme activity typically obtained, which increases the production costs and has a limited effect on food proteins. In industry, to benefit from the application of MTGase, an inexpensive source of MTGase is indispensable [26]. Consequently, it is critical to provide a high yield, high activity, and food grade (therefore safer) engineered MTGase. *B. subtilis* was the first prokaryotic bacteria that was found to contain MTGase, subsequently, the activity of MTGase was also detected in other bacteria, such as *B. circulans* BL32 [27,28]. Here, MTGase from *B. amyloliquefaciens* BH072 (BaMTGase) was chosen to express heterologously.

In this work, pNZ8048-SP*usp45-pro-Bamtg* was constructed and introduced into *L. lactis* NZ9000 via electrotransformation. It was demonstrated that, in the NICE expression system, the pro-BaMTGase was transcribed, translated, and secreted successfully in a relatively short time with the assistance of the Sec secretion system used by the SPUsp45. After optimization, the protein was obtained at a concentration of 48.7 ± 0.2 mg/L with an enzymatic activity of 28.6 ± 0.5 U/mg. Compared with MTGase which was heterologously expressed in other bacterial or yeast strains (unmutated), the purification process of BaMTGase in *L. lactis* was simplified, fermentation time was shorter, and the enzymatic activity was higher Table 3.

Research has shown that the center of the MTGases has the highest surface homology. In the structure of several MTG enzymes analyzed, the structural alignment of the catalytic triad (Cys-His-Asp) is similar, and although these three amino acids have different positions in the proteins, they are equivalent. However, significant differences among MTGases from different sources also exist, the higher activity of BaMTGase quantified in this work suggests that easier and more efficient purification methods such as the one developed here provide purer enzymes [29].

**Table 3:** Comparison of heterologous expression MTGases from different sources.

Source of MTGase	Expression Host Strain	Activity	Ref
*Streptomyces mobaraensis*	*Escherichia coli*	23 U/mg	[30]
*Streptomyces fradiae*	*Pichia pastoris* strain GS115	0.7 U/mL	[31]
*Streptomyces mobaraensis*	*Bacillus subtilis*	16.1 U/mg	[32]
*Streptomyces netropsis*	*Escherichia coli*	18.2 U/mg	[33]
*B. amyloliquefaciens* DSM7	*Escherichia coli*	37 mU/mg	[34]
*Streptomyces hygroscopicus* WSH03-13	*Yarrowia lipolytica*	5.3 U/mL	[35]
*Streptomyces mobaraensis*	*Bacillus subtilis*	29.6 U/mg	[14]
*Streptomyces mobaraensis*	*Lactococcus lactis* NZ9000	27.6 U/mg	[15]

Bacteria are shown in italics.

In the study of SPI solution crosslinking, the SDS-PAGE demonstrated that the enzymatic activity of recombinant BaMTGase was higher than that of commercial MTGase visually. The strength and WHC are the key factors to evaluate the properties of gels. In this work, recombinant BaMTGase was applied to the preparation of SPI gels, the textures and WHC of the BaMTGase-induced gels were stronger than that of the commercial MTGase crosslinked gels, indicating that the gel network was tighter and many macromolecular aggregates existed in BaMTGase-induced SPI gel, uniform gel network could lock water molecules in the gel, thus improved gel strength and WHC properties. The shape of BaMTGase-induced gels had almost no changes after squeezing through the texture analyzer, while the gels catalyzed by commercial MTGase showed obvious morphological changes after extrusion, and the initial morphology was unrecoverable after the measurement (Figure 4f–h). Compared with the protein secondary structure of commercial MTGase-induced gel, the BaMTGase-induced SPI gel exhibited an increase in *β*-sheet and *β-*turn and led to a reduction in *α*-helix and random coils. According to previous research, the *β*-sheet is predominant in the gel, the increase in *β*-sheet was common during protein gelation because hydrophobic groups within the protein are crosslinked into dense structures by MTGase induced and more aggregation of the protein occurred, whereas, the content of *α*-helix and WHC were negatively correlated, in BaMTGase-induced gel, the hydration of protein molecules with water was enhanced, reducing the hydrophobic effect of the protein. The results were correlated with the microstructure and texture of the gel [36,37,38].

The MTGase-induced gel microstructure is built as a three-dimensional network of proteins. The commercial MTGase-induced gels have a discontinuous and rough structure, where the connections between the holes are thin and soft, favoring the gel collapse upon mechanical stress. However, the recombinant BaMTGase-treated gel has more homogeneous and dense pores, and the connections between pores are smooth and robust. The results showed that the physical properties of SPI gels prepared with a small amount of BaMTGase were better.

## 3. Conclusions

In summary, the recombinant BaMTGase secreted by *L. lactis* NZ9000 is suitable for application in various industrial scenarios. This work provides a good cell factory (GRAS) for high-yield and high-quality BaMTGase industrial production. The changes induced in SPI gel properties also provide a positive theoretical basis for the application of BaMTGase to improve the physicochemical properties of food products that can greatly reduce production cost.

## 4. Materials and Methods

### 4.1. Materials, Bacterial Strains and Growth Conditions

M17 broth was offered by Haibo Biotechnology Co., Ltd. (Qingdao, China), Luria-Bertani (LB) broth, Chloramphenicol, and Soy protein isolate (SPI, ≥85% protein) were purchased from Solarbio Science & Technology Co., Ltd. (Beijing, China). Commercial MTGase was supplied by Yiming Biological Co., Ltd., (Jiangsu, China, 1000 U/g). The enzymes (Restriction enzymes, T4 ligase, etc.), kits, and protein markers were bought from NEB (Beijing, China). Chemical reagents, synthesis of oligonucleotides, and DNA sequencing services were provided by Sangon Biotech Co., Ltd. (Shanghai, China).

*B. amyloliquefaciens* BH072 was cultured in LB broth at 37 °C, 200 rpm. *Lactococcus strains* (*L. lactis* NZ9000 and *L. lactis* NZ9000 harboring pNZ8048-SP*usp45-promtg*) were grown in M17 medium supplemented with 0.5% glucose (GM17) at 30 °C. When pNZ8048 plasmid or its derivatives were present, chloramphenicol at a final concentration of 5 μg/mL was added to the GM17 medium.

### 4.2. Construction of Transformants

Molecular cloning and molecular manipulations were performed according to Sambrook, et al. [39]. The strains, vectors, and primers used to construct the recombinant expression system are summarized in Table 4. Primers P1 and P2 were used to amplify the *spusp45-proregion* from pNZ8048-SP*_usp45_-promtg* with pfu enzyme (NEB, Beijing, China) and the annealing temperature was 57 °C [15]. Primers P3 and P4 (61.2 °C) were used to amplify the *mtg* gene (GenBank: CP009938.1) from the genomic DNA of *B.* *amyloliquefaciens* BH072 (*Bamtg-6his*) and fused to the segment *usp45-pro* by splice overlap extension PCR. The fused fragment was amplified by P1 and P4 (60 °C) to obtain the final construct *spusp45-pro-Bamtg*. This and plasmid pNZ8048 were digested with *Nco*I and *Kpn*I and ligated using T4 DNA ligase to produce pNZ8048-SP*_usp45_-pro-Bamtg* plasmid, the construction progress of plasmid was shown in Figure S1. This plasmid was transformed into *L. lactis* NZ9000 for further characterization.

### 4.3. Recombinant BaMTGase Overexpression and the Optimization of Secretion Conditions in L. lactis NZ9000

The fermentation conditions for *L. lactis* NZ9000 (pNZ8048-SP*_usp45_-pro-Bamtg*) were optimized in GM17 broth with 5 μg/mL chloramphenicol. The fermentation time was firstly optimized, inoculating the strain into 100 mL GM17 at a ratio of 1:100 from a stationary phase culture. When the optical density (OD) 600 reached 0.6, 1 ng/mL nisin was added to induce the transcription of recombinant BaMTGase. Subsequently, five samples were collected after 8 h, 12 h, 24 h, 36 h, and 48 h at 30 °C. The supernatant was collected after centrifugation at 6000× *g* 15 min, 4 °C for further purification and analysis.

The concentration of the inducer was optimized after fermentation time optimization. Five cultures were prepared as indicated above. Nisin was added at OD_600_ 0.6 at different concentrations (1/3/5/7/9 ng/mL), and then fermentation continued for another 36 h. Then, the supernatant was collected for protein purification and analysis. Afterward, we measured the growth curves of the strains (*L. lactis* NZ9000 with no plasmid and *L. lactis* NZ9000 with plasmid pNZ8048 were considered as control) under the optimal conditions of protein secretion.

### 4.4. Protein Purification and Determination of Enzyme Activity

Nickel-nitrilotriacetic acid (Ni-NTA, Sangon Biotech Co., Ltd., Shanghai, China) resin was used according to the manufacturer to bind histidine-tagged target protein (BaMTGase, Protein ID: AJE79797.1). The supernatant was mixed with Ni-NTA resin for 2 h at 4 °C to allow protein binding. Then, pro-BaMTGases were recovered with an Elution buffer containing imidazole (1× column volume). Imidazole was removed through dialysis in 0.2 M PBS (pH 7.2) using <10 kDa membranes. After dialysis, the protein samples were stored at −80 °C. The purified protein was analyzed by SDS-PAGE [42]. MTGase was synthesized as an inactive zymogen, to active the zymogen, the proregion needed to be proteolyzed, hence, 1 mL Trypsin (10 mg/mL) was added to 50 mL supernatant before purification and the mixture was incubated at 37 °C for 1 h.

Protein concentration was measured with a commercial BCA kit (EasyII Protein Quantitative kit, TransGen, Beijing, China). The optical density was measured at 595 nm on a UV-Visible spectrophotometer (Shanghai Spectrum Instruments Co., Ltd., Shanghai, China), to calculate the protein concentration.

The enzymatic activity of BaMTGase was evaluated according to the classical colorimetric hydroxamate method with minor modifications [16]. Briefly, 0.5 mL BaMTGase solution (46.9 mg/L) was mixed with 0.9 mL substrates (30 mM Z-Gln-Gly, 10 mM glutathione, 100 mM hydroxylamine and 0.2 M Tris-HCl buffer, pH 6.0) and incubated at 37 °C for 10 min. After that, 2 mL of ferric chloride trichloroacetic acid reagent (12% trichloroacetic acid and 5% FeCl_3_·6H_2_O) were added to terminate the reaction immediately. The reaction system was centrifuged at 4000× *g* for 1 min and the absorbance of the supernatant was measured at a wavelength of 525 nm using a Spectrophotometer (Multiskan SkyHigh, Thermo Fisher Technology Co., Ltd., Waltham, MA, USA). Calibration curves were prepared with L-glutamic acid γ-monohydroxamate. A unit of MTGase activity was defined as the amount of MTG required to catalyze the formation of 1 *μ*mol L-glutamic acid γ-monohydroxamate per minute at 37 °C [14].

### 4.5. SPI Gelation Crosslinked by MTGase (SDS-PAGE)

SPI is a by-product of soybean processing, the protein content was high, containing a complete range of amino acids with nutritional value, in addition, SPI has diverse processing characteristics, including solubility, gelation, and foaming, hence, there are extensive applications in the food industry [43,44]. A 1% (*w*/*v*) SPI solution was prepared with distilled water, the insoluble fraction was removed by centrifugation at 10,000× *g* for 10 min. The solution was mixed with 0.05% (*w*/*v*) commercial MTGase or 0.05% (*w*/*v*) BaMTGase at a ratio of 10:1 (*v*/*v*) and incubated at 45 °C. Samples were collected after 15 min and 60 min crosslinking. Untreated 1% (*w*/*v*) SPI solution was considered as control. To investigate the applicability of BaMTGase, the covalent binding of SPI protein subunits after enzymatic crosslinking was monitored by SDS-PAGE.

### 4.6. Preparation of SPI Gels with MTGase

10 g of SPI protein powder was dissolved in 100 mL distilled water (10% SPI solution, *w*/*v*) by stirring at room temperature. 1 mL 300 mg/mL commercial MTGase or 1 mL 2 mg/mL BaMTGase was added to the SPI solution. The pH of the solution was adjusted to 7.0 with 0.05 M Tris-HCl buffer. After incubation in a thermostatic shaking water bath at 45 °C for 2 h, MTGase was inactivated heating at 95 °C for 10 min. Afterward, MTGase-induced SPI gels were cooled to room temperature and then stored in the refrigerator at 4 °C overnight for analysis or freeze-dried with a Freeze drier (Beijing Boyekang Experimental Instrument Co., Ltd., Beijing, China) for further use.

### 4.7. Determination of SPI Gel Texture

Gel texture was measured according to Gao et al. with some modifications [45]. The gel strength hardness, springiness, cohesiveness, and gumminess were measured using a TA-XT plusC texture analyzer (Stable Micro System Co., Ltd., Godalming, UK). After calibrating the instrument, the P/36R diameter probe was used to compress the gels (a cube with a volume of 1 cm^3^) at the center of the sample twice with 3 g trigger force, probe speed is 5 mm/s, test speed is 5.0 mm/s, and deformation is 50%. The gel strength was recorded as the maximum force generated press when the probe pressed against the gel deformation for the first time.

### 4.8. Water-Holding Capacity Determination

5 g of gel samples were weighted in a 10 mL tube and centrifuged at 7000× *g*, 4 °C for 20 min. Then, the water was removed carefully and the samples were weighed again. The water holding capacity (WHC) of the gel was calculated as follows:

$$\mathrm{WHC}=\frac{\left(Wt-Wr\right)}{Wt}\times 100\%$$

In this formula, *Wt* represents the total weight of water and *Wr* is the weight of water which was removed from the gel after centrifugation (g).

### 4.9. Fourier Transform Infrared Spectroscope (FT-IR) Analysis

The secondary structures of SPI gels were analyzed by using Nicolet 67 FT-IR spectrometer (Thermo Nicolet Inc, Waltham, MA, USA). The freeze-dried samples were ground into powder, and 0.1 g powder was placed on the ATR attachment. For each sample, a total of 64 scans were performed with a range from 550 to 4000 cm^−1^. The amide I band (1600–1700 cm^−1^) was analyzed by Peak Fit software (version 4.12, SPSS Inc., Chicago, IL, USA) to evaluate the changes of secondary structure in SPI gels, which related to the percentage of peak area of each spectrum.

### 4.10. Characterization of SPI Gel Microstructure

A field Emission Scanning Electron Microscope (FESEM, Hitachi, Regulus 8230, Japan) was used to explore the microstructure of SPI gel. Thinly sliced gel samples were freeze-dried and coated with gold, the sample was magnified 250 times and operated at a 2 kV accelerating voltage.

### 4.11. Protein Band Analysis and Statistical Analysis

Protein bands were analyzed in a semi-quantitative manner with the BANDSCAN software (Glyko Co., Ltd., Chicago, IL, USA). All experiments were conducted in triplicate, and the results are presented as the mean and standard deviation. The statistical analysis was performed with SPSS 19.0 (*p* < 0.05).

## Figures and Tables

**Table 1:** Texture of MTGase-induced SPI gels.

Sample	Enzyme Dosage (mL)	Hardness (g)	Springines-s (mm)	Cohesivene-ss (-)	Gumminess (g)
commercialMTGase	1	14.78 ± 0.7 ^b^	0.74 ± 0.08 ^b^	0.39 ± 0.03 ^b^	14.17 ± 1.03 ^b^
BaMTGase	1	108.33 ± 0.6 ^a^	1.80 ± 1.20 ^a^	0.81 ± 0.02 ^ab^	192.71 ± 3.88 ^a^

Values in the table are the mean ± standard deviation of three parallel experiments. Letters within each column indicate significant differences (*p* < 0.05). Letters within each column indicate significant differences (*p* < 0.05).

**Table 2:** Secondary structure constituents of samples.

	Content of Secondary Structure (%)			
Sample	*α*-Helix	*β*-Sheets	*β*-Turns	Random Coils
commercialMTGase	16.91 ± 0.69 ^a^	51.79 ± 0.75 ^b^	13.65 ± 0.49 ^b^	17.65 ± 0.58 ^a^
BaMTGase	15.02 ± 1.01 ^b^	53.1 ± 0.24 ^a^	16.40 ± 0.52 ^a^	15.48 ± 0.21 ^b^

Values in the table are the mean ± standard deviation of three parallel experiments. Letters within each column indicate significant differences (*p* < 0.05).

**Table 4:** Strains, vectors and primers used in this work.

Strain	Characteristic	Information
*Bacillus amyloliquefaciens* BH072	Amplification of *mtg*	Lab collection [40], BaMTGase wild-type producer
*Lactococcus lactis* NZ9000	heterologous host	*pepN::nisRK* [41]
Plasmid	Characteristic	Information
pNZ8048	P*_nisA_* promoter, Cm^R^	Inducible plasmid for NICE expression
pNZ8048-SP*usp45-promtg*	Amplification of *spusp45-proregion* gene, Cm^R^	Lab collection [15]
pNZ8048-SP*usp45-proBamtg*	Recombinant expression plasmid, Cm^R^	This work
Primer	Sequence (5′–3′)	Property/Function
P1	CATGCCATGGCAAAAAAGATTATCTCAGCTATTTTAAT	Amplification of the SP*_usp45-proregion_*which preceded by *Nco*I restriction site
P2	TGGCCGGATATGATAATCAT*GGGGGCCCGGA*	Amplification of the SP*_usp45-proregion_*, P2 and P3 complement in reverse
P3	*TCCGGGCCCCC*ATGATTATCATATCCGGCCA	Amplification of *mtg* gene, P3 and P2 are reverse complementary sequences
P4	GGGGTACCTTAGTGATGGTGATGGTGATGATGCATGATCTGATAAAGCG	Amplification of *mtg* gene. Hexahistidine codons were followed by a *Kpn*I restriction site

Cm^R^ chloramphenicol resistance. Restriction sites sequences introduced in the primers are underlined. Gene and the name of restriction sites are indicated in italics.

## Data Availability

Data is available on request from the authors.

## Supplementary Materials

The following supporting information can be downloaded at: https://www.mdpi.com/article/10.3390/gels8100674/s1, Figure S1: The construction progress of plasmid; Figure S2: The growth curves of strains.
