# Supplementary material for: Secretion of Bacillus amyloliquefaciens Transglutaminase from Lactococcus lactis and Its Enhancement of Food Gel Properties
Source: Gels. 2022 Oct 20;8(10):674. doi: 10.3390/gels8100674 (PMC9601987; doi:10.3390/gels8100674)
Supplement: Supplementary file 1 [file gels-08-00674-s001.zip › gels-1968851-supplementary.pdf]

Supplementary Materials

Secretion of *Bacillus amyloliquefaciens* Transglutaminase from *Lactococcus lactis* and Its Enhancement of Food Gel Properties

Tiange Ma [1], Xingjiang Li [1], Manuel Montalbán-López [2], Xuefeng Wu [1], Zhi Zheng [1,*] and Dongdong Mu [1,*]

[1] Anhui Fermented Food Engineering Research Center, School of Food and Biological Engineering, Hefei University of Technology, Hefei 230601, China

[2] Department of Microbiology, Faculty of Sciences, University of Granada, Granada 18071, Spain

* Correspondence: d.mu@hfut.edu.cn (D.M.); zhengzhi@hfut.edu.cn (Z.Z.)

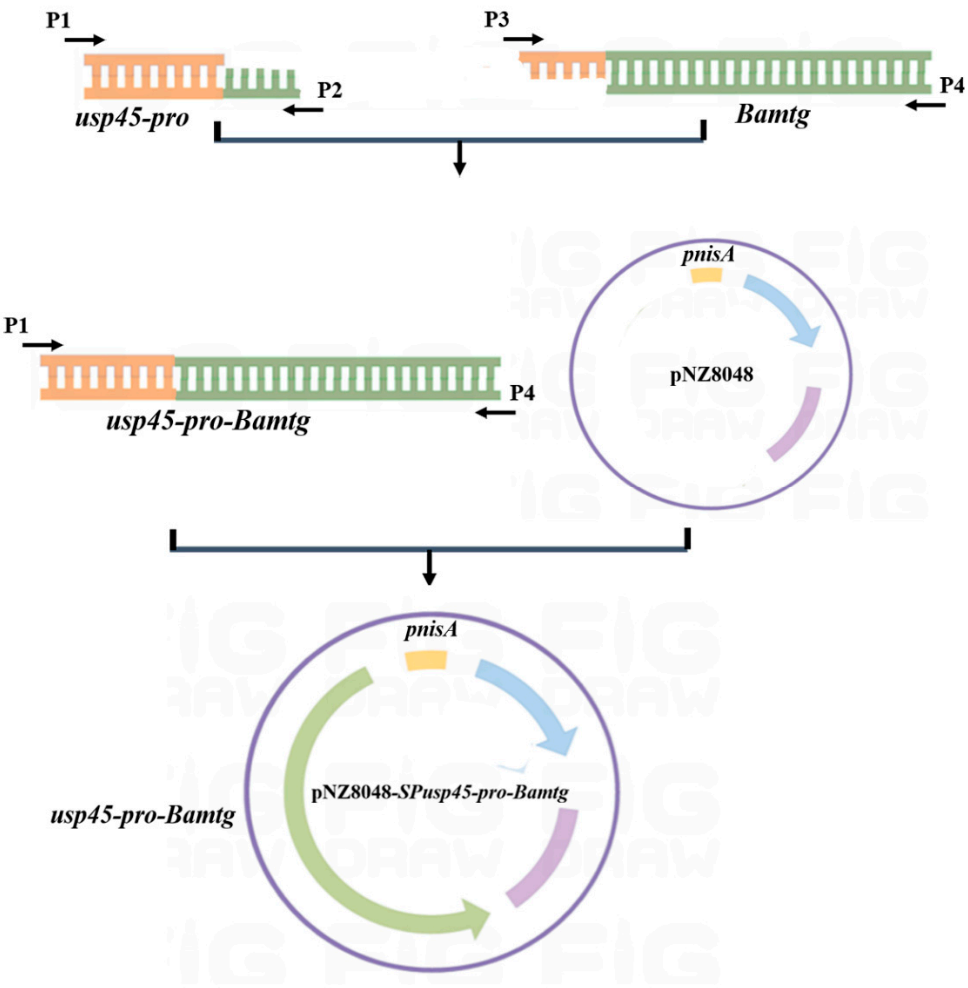

Figure S1. The construction progress of plasmid.

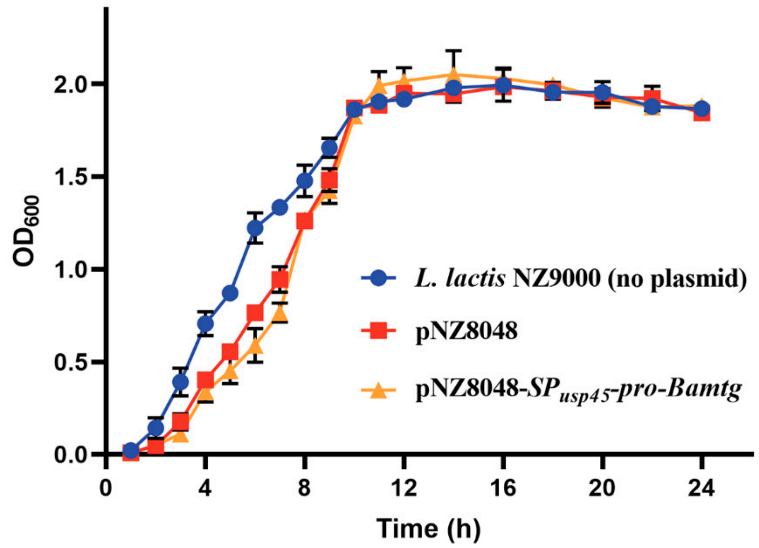

Figure S2. The growth curves of strains. Nisin was added when the OD is 0.6.
